# Supplementary material for: Flickering body temperature anticipates criticality in hibernation dynamics
Source: R Soc Open Sci. 2021 Jan 13;8(1):201571. doi: 10.1098/rsos.201571 (PMC7890501; doi:10.1098/rsos.201571)
Supplement: Supplementary material [file rsos201571supp1.pdf]

Flickering body temperature anticipates criticality in hibernation dynamics

DANIEL ORO[1] AND LÍDIA FREIXAS[2]

[1] Theoretical and Computational Ecology Laboratory, CEAB – Center for Advanced Studies (CSIC), Acces Cala Sant Francesc 14, 17300 Blanes, Spain

[2] Granollers Natural Sciences Museum, Francesc Macià 51, 08402 Granollers, Spain

Corresponding author:

Daniel Oro
Theoretical and Computational Ecology Laboratory
CEAB – Center for Advanced Studies (CSIC)
Acces Cala Sant Francesc 14
17300 Blanes, Spain
e-mail : d.oro@csic.es

Running head: Critical transitions in hibernation

Table S1 Details about how metric-based indicators of EWS of temperatures were applied and pre-processed to avoid biased results. TS = time series; T = temperature; BT = body temperature

Indicator	Statistical goal	Procedure for considering sensitivity to set up choices	Transformation	Filtering
BDS test	Detects non-linear serial dependence in the TS	We ran the test under different set ups for data transformation and filtering-detrending, because the test is sensitive to these choices	We log-transformed data (using log(T+1)) because of the presence of very fluctuating values between active BT (around 38º) and dormancy BT (close to 0º).	We filtered extreme data by fitting linear AR models, and we also generated partial autocorrelation functions for setting the best choice of time lags for the linear models fitted to the TS
Conditional heteroskedasticity (CH)	It measures whether variance at one time step is positively associated with variance at one or more previous steps; CH should increase when approaching a critical transition.	We used two lengths of rolling windows (10% and 30% of the total, the latter because of the slow timescale of varying temperatures in dormice). The significance threshold was set to 0.1	Data was standardized because of the presence of extreme values of BT in the dataset.	We filtered by fitting linear AR models
Nonparametric Drift-Diffusion-Jump (DDJ)	Models yield: conditional variance or drift, diffusion, jumps, and total variance		BT was log-transformed (using log(T+1))	
Generic early warning signals	EWS include: temporal autocorrelation of BT at lag-1, standard deviation SD and skewness	We used an overlapping moving window since indicators should be estimated as data become available	BT was log-transformed (using log(T+1)) and results were compared with raw BT	Data were filtered using Gaussian smoothing

Table S2 Partitioning of the times series and number of temperature records for each of the analyses.

Indicator	transition		Time series length (female/male)		
	To hibernation	To activity	To hibernation	To activity	After hibernation
BDS test	From start to first breaking point	From first to last breaking point	1124/1717	4498/3930	
Conditional heteroskedasticity	From start to first breaking point	From first to last breaking point	1124/1717	4498/3930	
Nonparametric drift-diffusion-jump models	Whole time series			8192	
AR(1), standard deviation, skewness	From start to first breaking point	From first to last breaking point	1124/1717	4498/3930	
	Idem adding 15 days of hibernation	Idem adding 30 days of activity	1502/2054	4682/4673	
Potential analysis	Whole time series			8192	
AR(p) of body temperature with air temperature	The whole series was partitioned in three periods: pre-hibernation, hibernation and post-hibernation (see electronic supplementary material, table S4)		1124/1717	4498/3930	2570/2545
Threshold AR(p) models	Whole time series			8192	

Table S3 BDS statistic on body temperature variability before the transition to hibernation (TH) and to activity (TA) for the male and female dormice for each model tested (first-difference, AR(1) and GARCH(0,1), three different values of ε (.5, .75 and 1, which were used to scale the standard deviation of the time series) and two embedding dimensions, D, up to which the test was performed. All statistical tests for detrended data (using 1000 bootstrapping iterations) were significant (*P* < 0.0001).

Model		D	First-difference detrending data			AR(1) residuals			GARCH(0,1) residuals		
ε value			0.5	0.75	1	0.5	0.75	1	0.5	0.75	1
Female											
	TH	2	15.40	17.18	18.74	18.62	18.85	19.31	27.40	29.26	26.46
		3	16.61	17.69	18.67	21.17	20.17	19.91	29.62	30.42	26.78
	TA	2	57.02	53.65	48.14	59.38	52.14	45.78	43.48	44.84	42.57
		3	64.12	54.41	47.60	66.12	53.47	45.32	52.45	48.66	42.94
Male											
	TH	2	24.06	24.26	23.73	33.89	21.53	16.28	44.16	41.23	34.88
		3	25.67	23.61	22.34	46.36	28.08	17.91	49.81	44.14	35.91
	TA	2	46.94	49.84	45.36	48.11	47.54	44.07	25.64	35.93	36.71
		3	50.23	49.36	44.70	52.09	47.62	43.28	32.36	39.23	36.16

Table S4 Coefficients of the parameters of regression AR(p) models of body temperature T, in which air temperature was added (A), compared to AR(p) models not including this covariate. All temperatures were standardized for each of the three periods considered: pre-hibernation, hibernation and post-hibernation (a, b and c respectively, see figure; note that periods a and b correspond to transition to hibernation (TH) and to activity (TA)). Models have the form: $T(t) = \beta A_i + \emptyset_0 + \sum_{i=1}^{p} \emptyset_i (T(t-i) - \emptyset_0)$, in which p = 1. Standard errors of the mean (SE) are shown in parentheses; ΔAIC = difference in AIC value with the best model in each of the comparisons. All models including air temperature as a covariate showed lower AIC values.

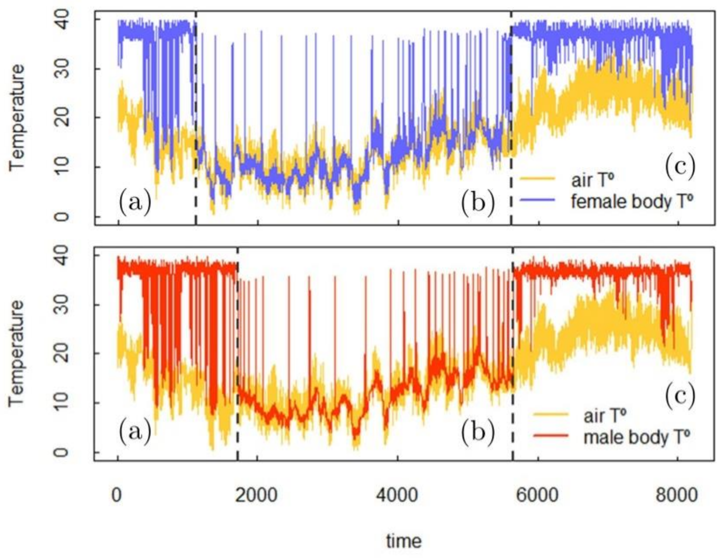

Dormice	Period	Air T (ºC)	Parameters			ΔAIC
			β	ϕ_0	ϕ_1	
female	Pre-hibernation (a)	Included	-.0320 (.043)	-.004 (.199)	.958 (.001)	0
		Not included		-.001 (.810)	.999 (NA)	26.45
	Hibernation (b)	Included	.069 (.012)	.001 (.105)	.964 (.004)	0
		Not included		.000 (.114)	.966 (.004)	13.81
	Post-hibernation (c)	Included	.148 (.031)	-.014 (.098)	.923 (.008)	0
		Not included		-.011 (.099)	.924 (.008)	20.91
male	Pre-hibernation (a)	Included	-.116 (.045)	.001 (.151)	.949 (.001)	0
		Not included		.000 (.142)	.945 (.001)	4.65
	Hibernation (b)	Included	.868 (.012)	-.001 (.001)	.477 (.021)	0
		Not included		.001 (.101)	.952 (.005)	731.16
	Post-hibernation (c)	Included	.107 (.032)	-.004 (.094)	.915 (.008)	0
		Not included		-.011 (.095)	.912 (.008)	9.28

Figure S1 Partial autocorrelation functions for the body temperature time series partitioned for the period before the transition to hibernation (panels a and b for the female and the male, respectively) and before the transition to activity (panels c and d for the female and the male, respectively). Body temperature when dormice were active and prior to hibernation (panels a and b for the female and the male respectively) showed a circadian cyclicity, but not during hibernation.

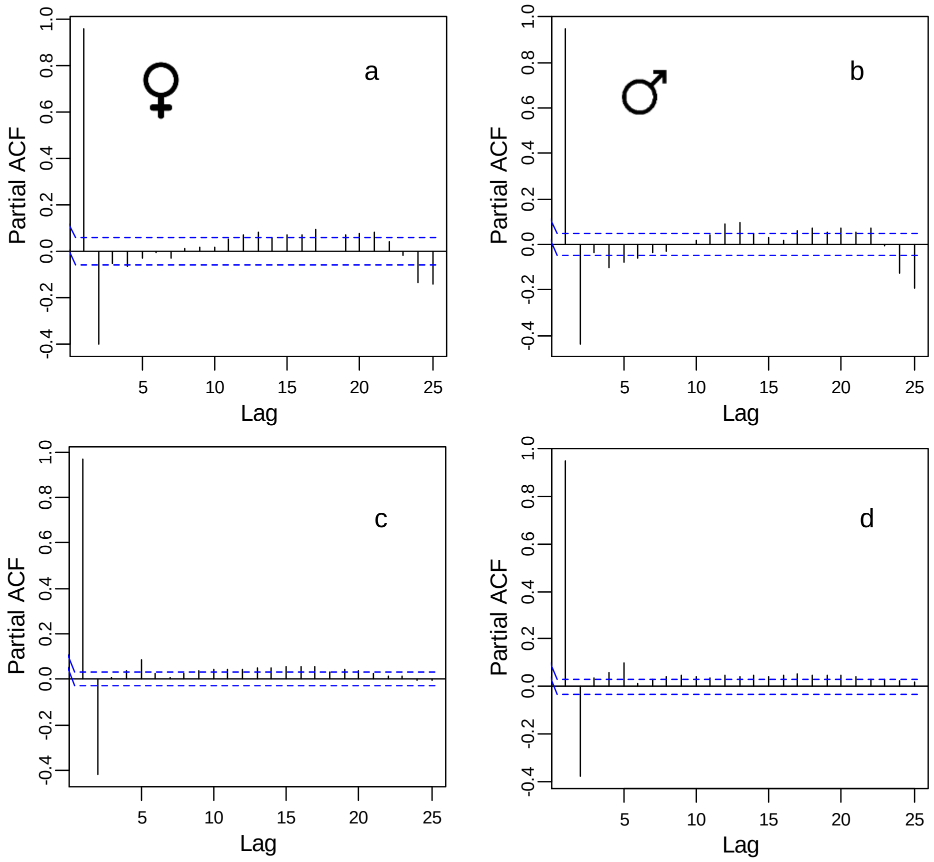

Figure S2 Results from conditional heteroskedasticity (expressed as the R^2 values of the regressed residuals) for the transitions to hibernation (panels (a) and (b) for female and male respectively) and transitions to activity (panels (c) and (d) for female and male respectively), considering two sizes of rolling windows (lengths 10% and 30% of the total). The red lines show the limit above which R^2 values are statistically significant (P = 0.1). CH was sensitive to rolling window size.

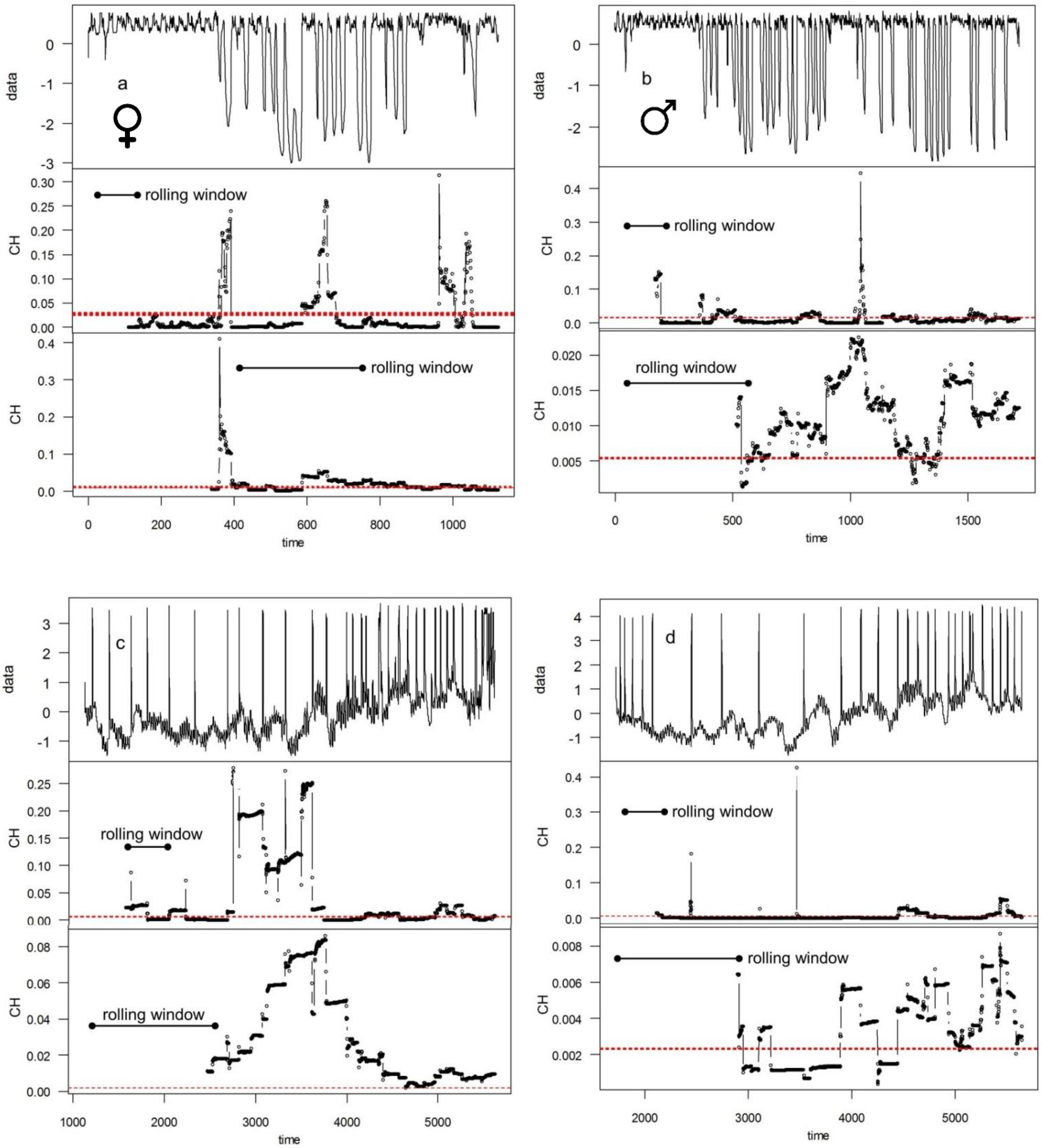

Figure S3 Nonparametric drift-diffusion-jump model-based indicators for body temperature dynamics of the female (panels a to e) and the male (panels f to j) edible dormouse. Left panels show (from top to bottom) how body temperature, conditional variance, diffusion, jump intensity and total variance vary with time, whereas right panels show how the same indicators vary with body temperature. Dashed lines show the two breaking points signalling transition to hibernation and activity. When plotted against body temperature, conditional variance peaked around value 3 (20.1ºC), which was the temperature that separates the two states of hibernation and activity, and jump intensity peaked at value 2.5 (12.2ºC), which measures the average jump in temperature from dormancy to T_e and vice versa.

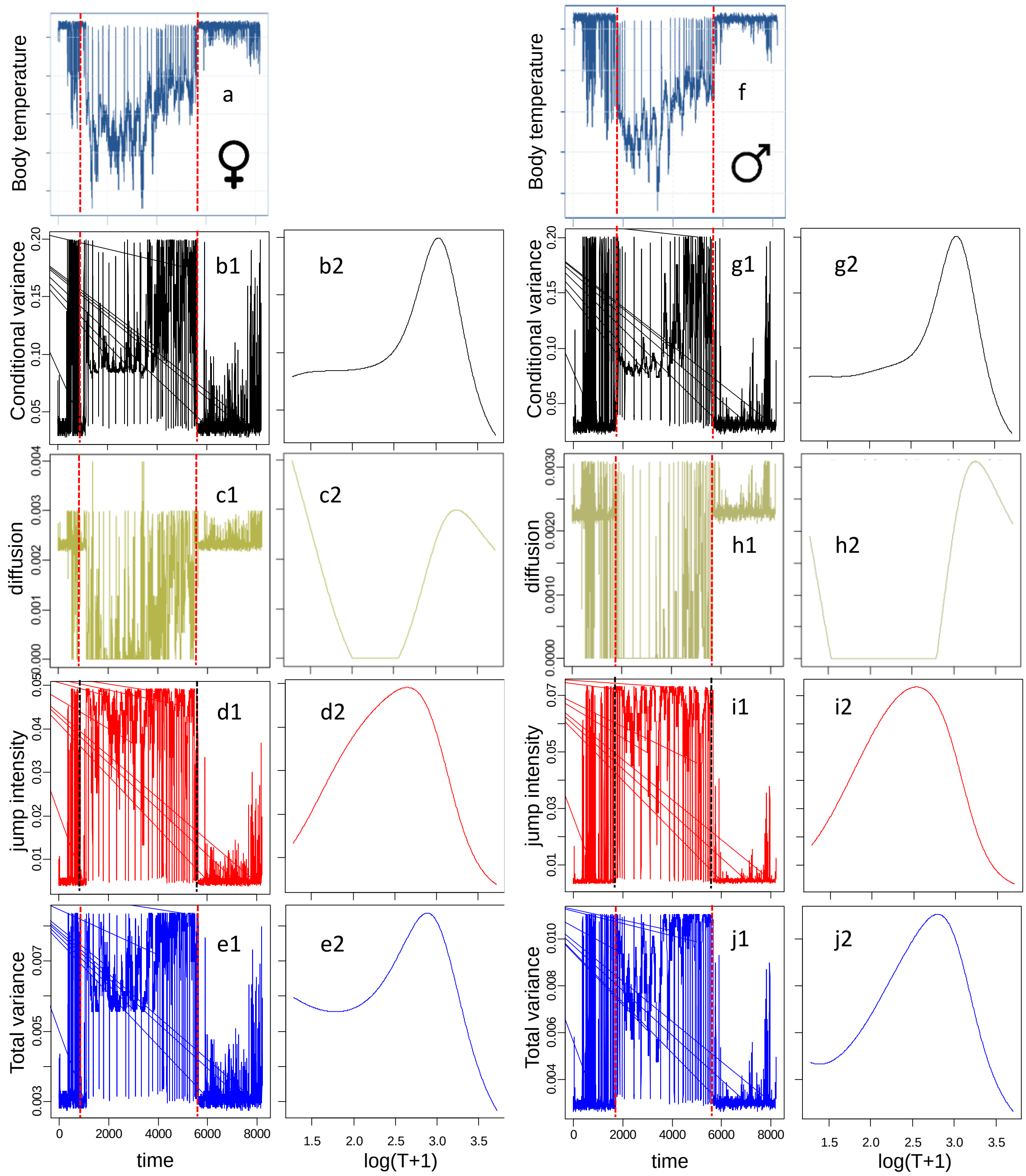

Figure S4 Metric-based rolling window indicators estimated for the body temperature time series for the two dormice and separated for each alternative state (activity and hibernation). For the transition to hibernation, we added 15 days of data once hibernation started (as marked by the first breaking point in the time series) and 30 days of data from the onset of activity (as marked by the last breaking point). (a and b): activity state and hibernation state for the female; (c and d): activity state and hibernation state for the male. Red lines show the Gaussian filtering of the time series. Panels 1, 2 and 3 show autocorrelation at lag-1 (AR(1)), standard deviation and skewness respectively, estimated within sliding windows of 20% the size of the time series. The Kendall τ indicates the strength of the trend in the indicators. Vertical dashed lines show the breaking points separating the two stable states.

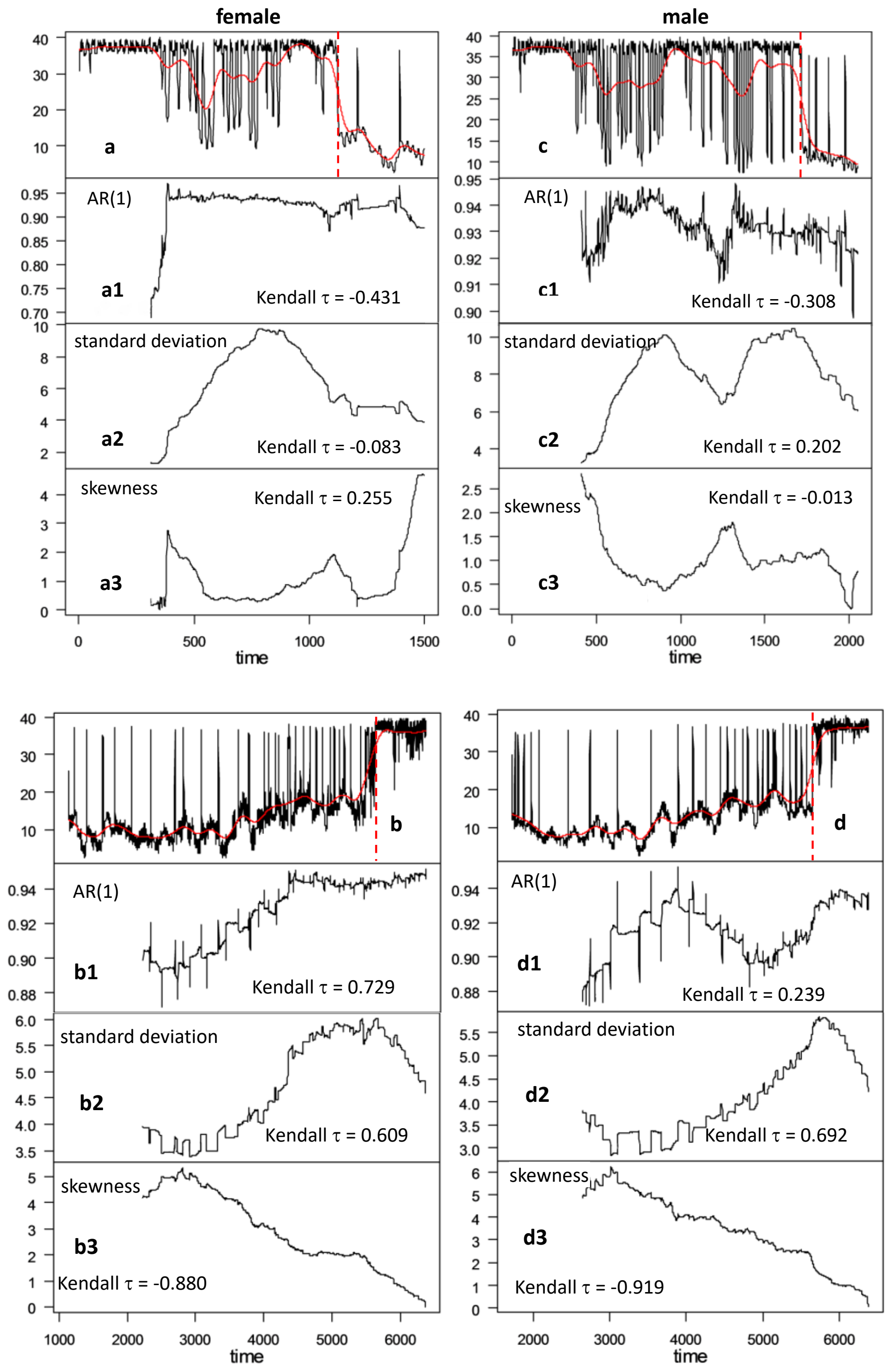

Figure S5 Metric-based rolling window indicators estimated for the body temperature time series for the two dormice for the whole time series (a and b for female and male, respectively). Red lines show the Gaussian filtering of the time series. Panels 1, 2 and 3 show autocorrelation at lag-1 (AR(1)), standard deviation and skewness respectively, estimated within sliding windows of 20% the size of the time series. The Kendall τ indicates the strength of the trend in the indicators. Vertical dashed lines show the breaking points separating the two stable states.

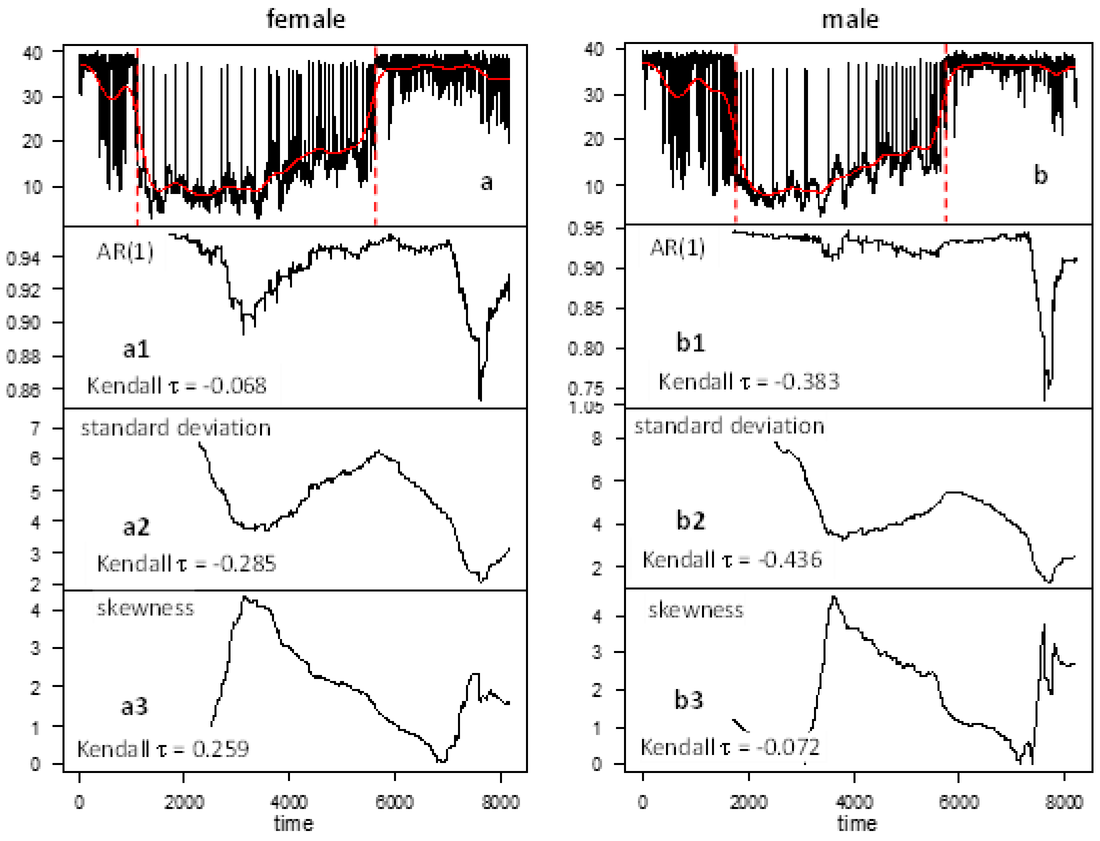

Figure S6 Metric-based rolling window indicators estimated for the body temperature time series and air temperature time series (yellow lines) for the two dormice and separated for each alternative state (activity and hibernation). (a and b): activity state and hibernation state for the female; (c and d): activity state and hibernation state for the male. Red lines show the Gaussian filtering of the time series. Panels 1, 2 and 3 show autocorrelation at lag-1 (AR(1)), standard deviation and skewness respectively, estimated within sliding windows of 20% the size of the time series. The Kendall τ indicates the strength of the trend in the indicators. Vertical dashed lines show the breaking points separating the two stable states.

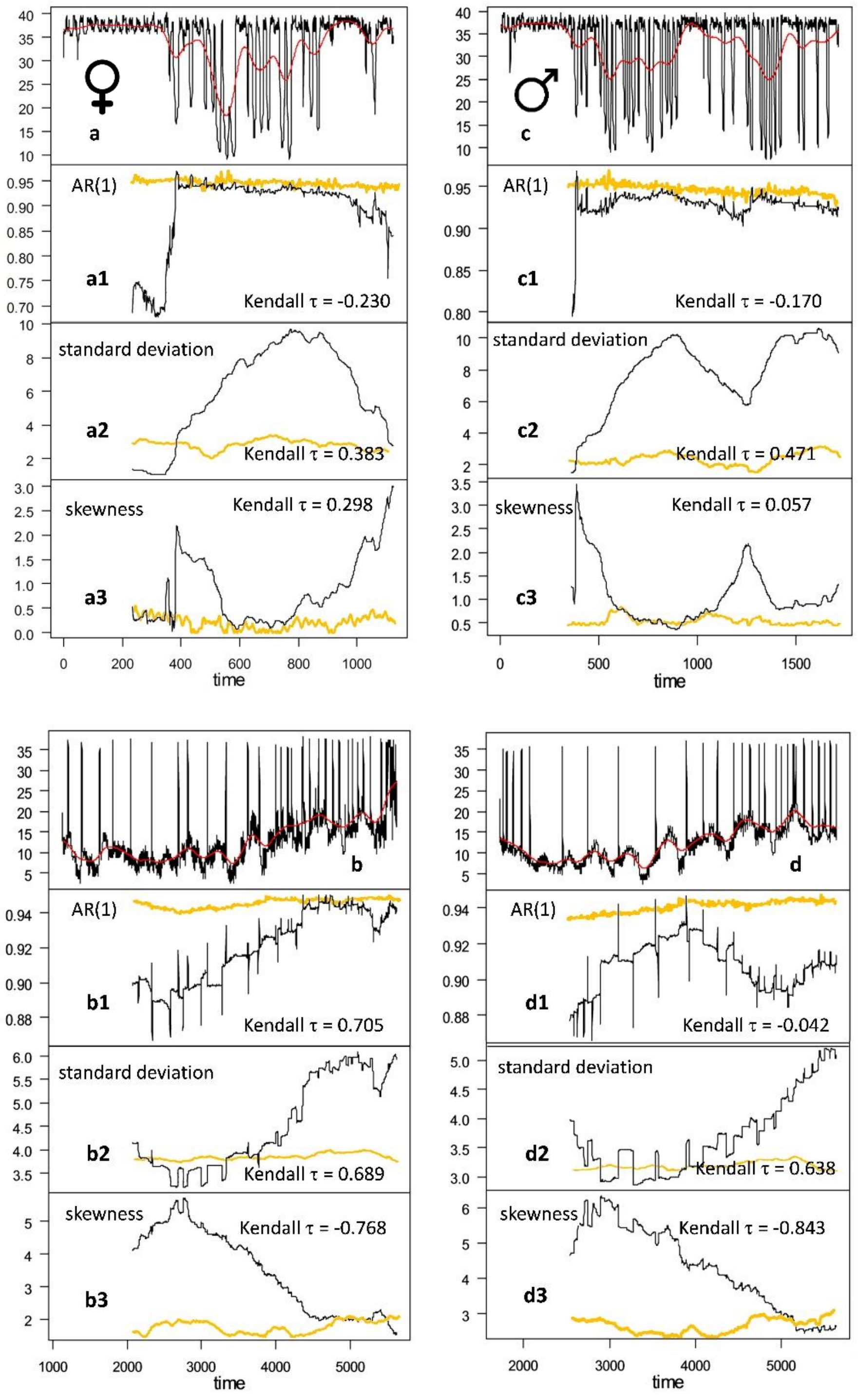

Figure S7 Sensitivities of generic EWS (autocorrelation at lag-1 AR(1), standard deviation SD and skewness Sk) for testing the robustness of the rolling window metrics. For each of the two transitions and each dormouse, left panels are the contour plots showing the influence of the width of the rolling window and Gaussian filtering on the observed trend in the metrics as measured by Kendall's τ; right panels are the histograms with the frequency distribution of the trend for τ. Small triangles show the parameter choice used in each of our analyses. Windows sizes ranged from 20% to 80% in increments of 10 points and bandwidths ranged from 5 to 200 in an increment of 20.

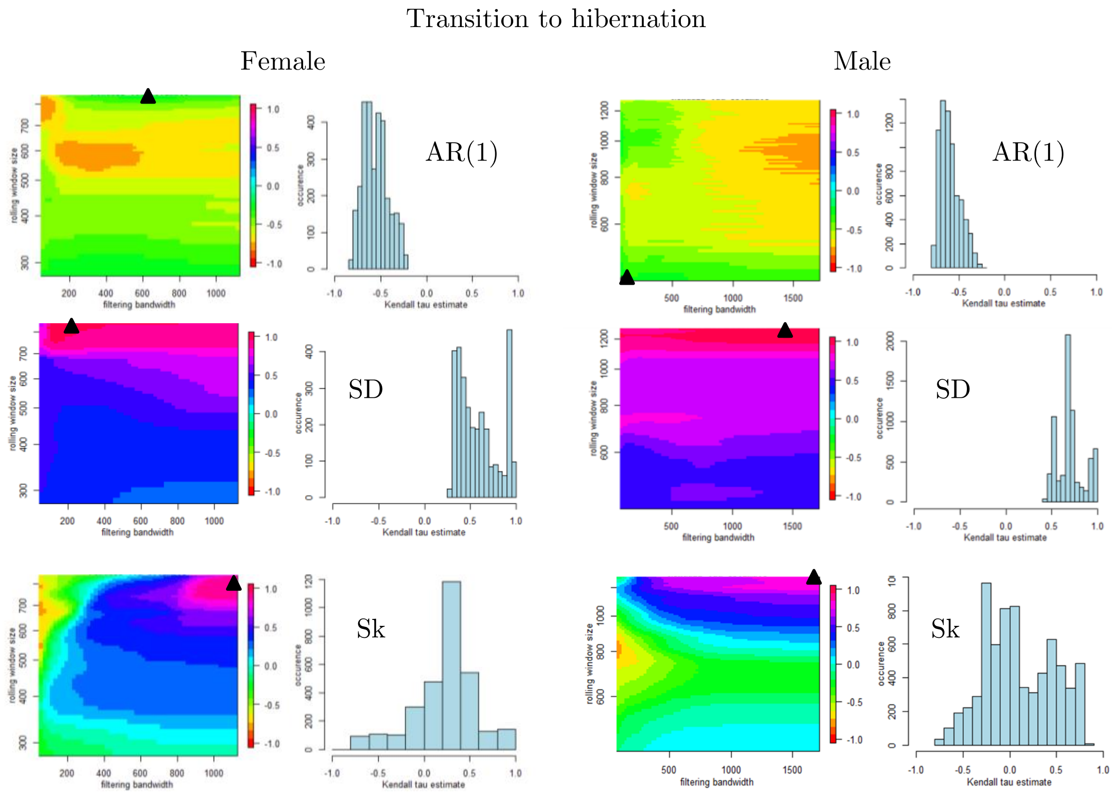

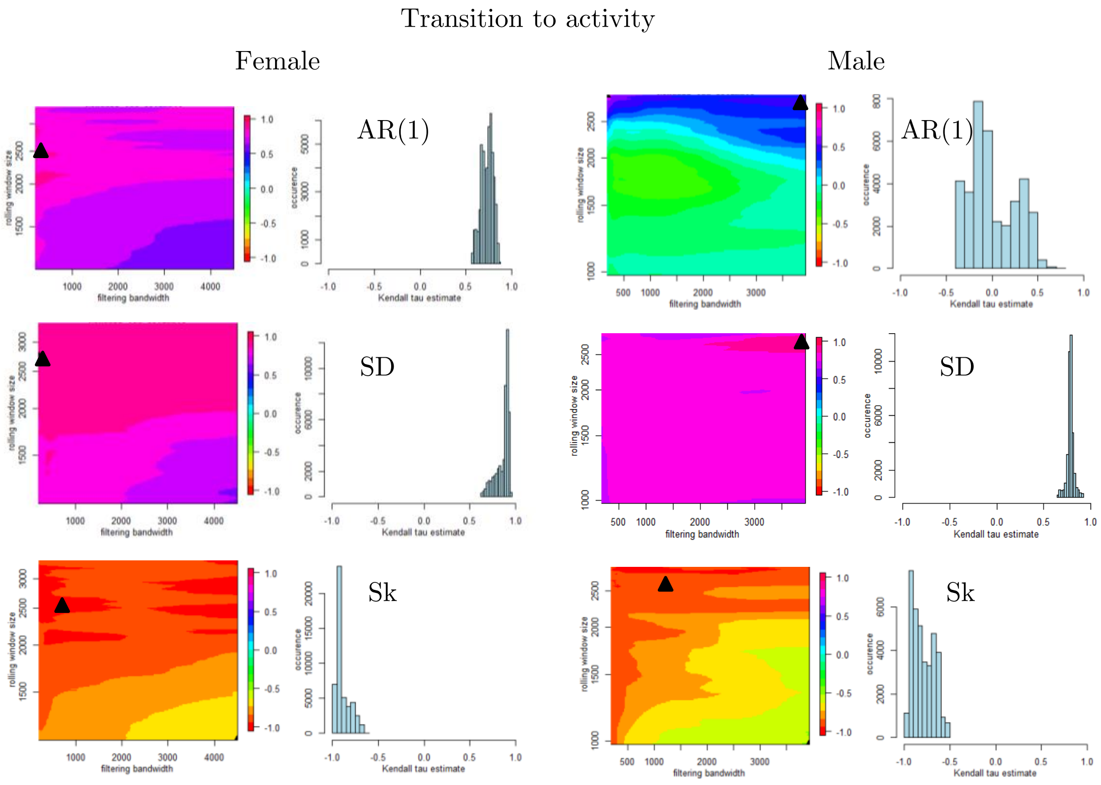
Transition to activity
Female
Male
AR(1)
SD
Sk
rolling window size
filtering bandwidth
occurence
Kendall tau estimate
